# Neural Response to Low Energy and High Energy Foods in Bulimia Nervosa and Binge Eating Disorder: A Functional MRI Study

**DOI:** 10.3389/fpsyg.2022.687849

**Published:** 2022-04-21

**Authors:** Brooke Donnelly, Nasim Foroughi, Mark Williams, Stephen Touyz, Sloane Madden, Michael Kohn, Simon Clark, Perminder Sachdev, Anthony Peduto, Ian Caterson, Janice Russell, Phillipa Hay

**Affiliations:** ^1^Clinical Psychology Unit, School of Psychology, University of Sydney, Camperdown, NSW, Australia; ^2^Peter Beumont Tertiary Eating Disorder Service, Department of Psychiatry, Royal Prince Alfred Hospital, Camperdown, NSW, Australia; ^3^School of Medicine, Western Sydney University, Campbelltown, NSW, Australia; ^4^Department of Cognitive Science, Macquarie University, Ryde, NSW, Australia; ^5^InsideOut Institute, University of Sydney, Camperdown, NSW, Australia; ^6^Department of Psychological Medicine, Sydney Children’s Hospital Network, Westmead Campus, Westmead, NSW, Australia; ^7^Faculty of Medicine and Health, University of Sydney, Camperdown, NSW, Australia; ^8^Department of Paediatrics and Child Health, Sydney Children’s Hospital Network, Westmead Campus, Westmead, NSW, Australia; ^9^Adolescent and Young Adult Medicine Unit, Sydney Children’s Hospital Network, Westmead Campus, Westmead, NSW, Australia; ^10^Centre for Research Into Adolescents' Health, Westmead Research Foundation, Westmead, NSW, Australia; ^11^Centre for Healthy Brain Ageing, Neuropsychiatric Institute, Prince of Wales Hospital, Randwick, NSW, Australia; ^12^Department of Radiology, Westmead Hospital, Westmead, NSW, Australia; ^13^Discipline of Medical Imaging, Charles Perkins Centre, Boden Institute, University of Sydney, Camperdown, NSW, Australia; ^14^School of Medicine, Translational Health Research Institute, Western Sydney University, Campbelltown, NSW, Australia

**Keywords:** fMRI, bulimia nervosa, binge eating disorder, female, emotions, foods

## Abstract

**Objective:**

Bulimia nervosa (BN) and binge eating disorder (BED) are eating disorders (EDs) characterized by recurrent binge eating (BE) episodes. Overlap exists between ED diagnostic groups, with BE episodes presenting one clinical feature that occurs transdiagnostically. Neuroimaging of the responses of those with BN and BED to disorder-specific stimuli, such as food, is not extensively investigated. Furthermore, to our knowledge, there have been no previous published studies examining the neural response of individuals currently experiencing binge eating, to low energy foods. Our objective was to examine the neural responses to both low energy and high energy food images in three emotive categories (disgust; fear; and happy) in BN and BED participants.

**Methods:**

Nineteen females with BN (*n* = 14) or BED (*n* = 5), comprising the binge eating group (BEG; *N* = 19), and 19 age-matched healthy control (HC)’s completed thorough clinical assessment prior to functional MRI (fMRI). Neural response to low energy and high energy foods and non-food images was compared between groups using whole-brain exploratory analyses, from which six regions of interest (ROI) were then selected: frontal, occipital, temporal, and parietal lobes; insula and cingulate.

**Results:**

In response to low energy food images, the BEG demonstrated differential neural responses to all three low energy foods categories (disgust; fear; and happy) compared to HCs. Correlational analyses found a significant association between frequency of binge episodes and diminished temporal lobe and greater occipital lobe response. In response to high energy food images, compared to HC’s, the BEG demonstrated significantly decreased neural activity in response to all high energy food images. The HC’s had significantly greater neural activity in the limbic system, occipital lobe, temporal lobe, frontal lobe, and limbic system in response to high energy food images.

**Conclusion:**

Results in the low energy food condition indicate that binge frequency may be related to increased aberrant neural responding. Furthermore, differences were found between groups in all ROI’s except the insula. The neural response seen in the BEG to disgust food images may indicate disengagement with this particular stimuli. In the high energy food condition, results demonstrate that neural activity in BN and BED patients may decrease in response to high energy foods, suggesting disengagement with foods that may be more consistent with those consumed during a binge eating episode.

## Introduction

Recurrent binge eating (BE) is a distressing symptom seen across the eating disorder (ED) diagnostic spectrum. Binge eating is a core diagnostic criterion for bulimia nervosa (BN) and binge eating disorder (BED); it also occurs in anorexia nervosa-binge purge type (AN-BP) and is a common feature in other specified feeding and eating disorder (OSFED). Binge eating involves either recurrent objective binge episodes (OBE), where the amount of food is definitely larger than what would usually be eaten for the social/cultural context, or subjective episodes wherein less food is consumed. Irrespective of the type of binge, the individual experiences a strong sense of a loss of control over the eating episode, which leads to ongoing psychological distress. For those with BN, binges are followed by inappropriate compensatory behaviors to avoid weight gain, such as purging, while BED involves engaging in recurrent binge episodes (BE) without compensation (APA, 2013). Considering the diagnostic overlap between eating disorder categories, with binge eating presenting a clinical feature that occurs transdiagnostically, it is appropriate to complete an investigation of this phenomenon in its own right.

Individuals with BN or BED commonly report consuming high energy, highly palatable, or pleasurable foods during a binge episode, likely to be linked to the caloric restriction that often precedes the binge episodes (Fairburn, 2008; Zunker et al., 2011). Research has consistently highlighted that people with a diagnosed ED or sub-threshold symptoms self-report higher negative emotions (e.g., disgust, anxiety, and fear) in response to food stimuli compared to healthy controls (HCs; Uher et al., 2003, 2004; Santel et al., 2006; Rodríguez et al., 2007; Foroughi et al., 2018). Harvey et al. (2002) found levels of disgust and fear in response to high-calorie food images increased with abnormal eating attitudes and in a later study of non-ED women, as eating concerns increased, so too did the degree of fear response to food images (McNamara et al., 2008). A recent study reported negative emotional responses to food images in ED participants were associated with a fear of a loss of control over ones’ eating (Hay and Katsikitis, 2014), one of the diagnostic features of BN and BED.

The neural reward system is known to contribute significantly to the drive to eat (Frank, 2013). Although the neural networks associated with eating and food-related stimuli have been examined less in BN and BED compared to anorexia nervosa (AN; Phillipou et al., 2014; Donnelly et al., 2018) a growing body of research suggests there exists an imbalance of the frontostriatal reward system and control-related circuit abnormalities believed to underlie binge eating (Appelhans, 2009; Frank, 2013; Berner and Marsh, 2014; Wierenga et al., 2014; Kessler et al., 2016; Lavagnino et al., 2016; McClelland et al., 2016). There is also evidence of the role of the cerebellum in feeding and appetitive eating patterns (Mahler et al., 1993; Zhu and Wang, 2008). A recent study highlighted the differential activity of this region in patients with BN compared to AN and healthy controls, with results demonstrating anterior cingulate cortex (ACC) hyperconnectivity to the cerebellum in bulimic participants (Amianto et al., 2013).

An increasing number of studies have examined neural responses specifically to food image stimuli in EDs. To date, studies have been small and findings inconsistent, leaving the neural underpinnings for recurrent binge eating unclear. A systematic review found that functional imaging studies showed decreased regulation in neural areas consistent with disengagement from food image stimuli and higher emotive involvement in people with an ED (Giel et al., 2011). A subsequent review examining neural responses to visual food cues concluded there are differences between people with an ED and healthy individuals in two brain circuits; (i) the limbic and para-limbic areas associated with salience and reward, and (ii) prefrontal region underlying cognitive control processes (Garcia-Garcia et al., 2013). In a recent systematic review of 32 neuroimaging studies in BN and BED, findings were mixed; both hyperactivity and hypoactivity in the neural reward and regulatory circuitry systems have been reported, alongside aberrant responding to disorder-relevant stimuli, including food images (Donnelly et al., 2018). One clear finding was that illness severity, defined by the frequency of bulimic or binge episodes, was related to greater aberrant neural activity compared to controls (Donnelly et al., 2018). In terms of studies specifically using food stimuli during functional MRI (fMRI), we found four such studies (Uher et al., 2004; Schienle et al., 2009; Brooks et al., 2011; Skunde et al., 2016), within which a number of regions were reported as showing aberrant activity: the frontal lobe, occipital lobe, insula, cingulate and temporal lobe. The parietal lobe may also demonstrate aberrant activity.

Overall there is evidence of a greater aversive and negative emotional response to food stimuli, abnormalities in reward processing; and emotional and behavioral dysregulation expressed *via* compulsive binge eating in BN and BED. Collectively, these findings support the hypothesis that aberrant neural responses will be found in people diagnosed with BN and BED compared to a HC group, in response to food image stimuli.

To our knowledge, no previous fMRI studies including BN and BED participants have separated pictorial food stimuli into low and high energy foods. The separation of food stimuli into these two categories offers a novel investigation and will provide clinically meaningful insight into whether high energy foods, associated with binge episodes, prompt a differential neural response related to low energy foods. In the present paper, we will therefore examine the neural response during fMRI to the low energy food stimuli and high energy food stimuli separately, with the results pertaining to each food image category analyzed separately, with a broader consideration of results across low and high energy conditions, made in the discussion.

The overall aim of this study is to investigate differences in neural response to both low energy food images and high energy food images, compared to emotionally neutral images, between individuals with BN or BED [binge eating group (BEG)] and a HC group, using fMRI. As this is a novel study, we will report whole-brain exploratory analyses in addition to region-of-interest (ROI) effects. Following on from the studies described above, we hypothesized there would be differential neural activity between groups in the frontal lobe, occipital lobe, temporal lobe, parietal lobe, insula, and cingulate. Due to a previous systematic review (Donnelly et al., 2018), highlighting the mixed findings in the neuroimaging profile of individuals who binge eat, we are predicting aberrant neural activity in the aforementioned neural regions, rather than specific hyper- or hypoactivity. We will also examine the relationship between neural activity in response to food images and illness severity (frequency of binge episodes and scores on measures of eating disorder pathology) using beta value correlations.

## Materials and Methods

### Participants

Nineteen females with a DSM-5 diagnosis of BN (*n* = 14) and BED (*n* = 5) and 19 age-matched HC female participants completed the study. Diagnosis was established by clinical interview by experienced eating disorder clinicians. For the present study, the BN and BED groups were analyzed as one group, named the BEG. This decision was made due to the primary diagnostic feature of the eating disorder–binge eating episodes. AN-BP was excluded from this study due to the confounding impacts of malnutrition of imaging results. Further to this, independent samples *t*-test analyses of the BN and BED groups were conducted and found no statistically significant differences between the groups on body mass index (BMI; weight in kg, divided by height in m^2^) or other clinical data. Additionally, this decision meant that the sample exceeded the minimum power requirements of the study (Desmond and Glover, 2002).

Eating disorder participants were recruited from hospital ED clinics and community advertising in Sydney between 2008 and 2015. HC participants were recruited from community media advertising. HCs were medically stable with no history of an ED and no current mental health diagnosis. Complete participant information is presented in Table 1.

**Table 1:** Participant information and assessment scores.

	BEG *N* = 19 (BN *n* = 14; BED *n* = 5)		HC *N* = 19		Group differences
	Mean	SD	Mean	SD	*p*
Age	26.63	13.04	21.74	1.15	0.136
BMI at assessment	25.04	6.35	22.98	4.97	0.270
**EDE-Q**					
Global	4.10	1.15	1.17	0.96	<0.001
Restraint	3.32	1.64	0.89	0.93	<0.001
Shape concern	4.73	1.19	1.62	1.54	<0.001
Weight concern	4.57	1.09	1.52	1.49	<0.001
Eating concern	3.89	1.32	0.67	0.79	<0.001
OBE frequency past 28 days	6.32	6.92	0.56	0.24	<0.001
SBE frequency past 28 days	7.84	9.45	0.68	1.56	<0.001
**HADS**					
HADS depression	9.26	3.89	5.21	4.52	0.005
HADS anxiety	12.47	4.31	5.26	2.90	<0.001
**TAS**					
Identifying	24.95	6.47	13.89	6.37	<0.001
Describing	16.68	4.50	12.47	4.64	0.007
Externalizing	21.05	4.77	18.73	4.02	0.115
TAS all	62.68	13.77	45.11	12.94	<0.001
EDI—body dissatisfaction	19.95	4.64	7.00	7.55	<0.001
Satiety pre-fMRI	5.73	3.07	3.65	2.19	0.059
Satiety post-fMRI	4.77	1.78	3.18	2.37	0.065

BMI, body mass index; SD, standard deviation; BEG, binge eating group comprising bulimia nervosa (BN; *n* = 14) and binge eating disorder (BED; *n* = 5); HC, healthy controls; *p*, probability; OBE, objective binge episode; SBE, subjective binge episode; EDE-Q, Eating Disorder Examination-Questionnaire; HADS, Hospital Anxiety and Depression Scale; TAS, Toronto Alexithymia Scale; and EDI, Eating Disorder Inventory.

Initial individual screening was completed on enrolment to the study based on the exclusion criteria described above by research Psychologists, either in person in a clinical setting or over the phone for those recruited in the community. Those meeting criteria for the study were offered an in-person appointment to complete the full assessment battery, with either an experienced Clinical Psychologist or Psychiatrist. In addition, height and weight measurements to one decimal place were taken. Weight was taken in light clothing. A separate appointment was subsequently scheduled for the fMRI scan.

Exclusion criteria were: a history of head trauma, hearing or visual impairments, neurological conditions or diseases, pacemakers, defibrillators, artificial heart valves, stents, neurostimulators, cochlear or stapes implants, metallic implants, cerebral aneurysm clips, pregnancy, claustrophobia, a current or past diagnosis of post-traumatic stress disorder or schizophrenia. All participants spoke English.

Participants gave written informed consent. In exchange for any associated travel costs to attend the assessment and fMRI appointments, participants were reimbursed with a $30 gift voucher.

### Sample Size

The published data and guidelines of Desmond and Glover (2002) to estimate sample size in fMRI neuroimaging studies, indicate for a liberal threshold of 0.05, 12 participants per group is required to achieve 80% power at the single voxel level. Therefore, we estimated that the sample of 19 participants in the BEG and HC group, respectively provides robust power.

### Assessment Battery

#### Neuropsychiatric Assessment

The Mini International Neuropsychiatric Interview-Plus (MINI-P, version 5; Sheehan et al., 2005) was completed with all participants to screen for Axis I disorders, based on the DSM-5. The MINI-P was also used to confirm eating disorder diagnoses. For the present study, a diagnosis of BN or BED was required for inclusion.

#### Eating Disorder Symptoms

The Eating Disorder Examination-Questionnaire (EDE-Q; Beglin and Fairburn, 1992) was used to assess ED symptoms over the preceding month. The EDE-Q focuses on the previous 28 days and eating disorder behaviors are assessed in terms of the number of symptom episodes, for example, binge episodes, occurring during this period. Subscale scores (restraint, eating concern, weight concern, and shape concern) and a global score may be derived from the 29 items addressing attitudinal aspects of eating disorder psychopathology. Mond et al. (2006) have published Australian community normative data for the EDE-Q subscales. The EDE-Q has sound internal consistency, test–retest reliability, and temporal stability.

#### Body Dissatisfaction

Body dissatisfaction was assessed using the nine-item body dissatisfaction subscale from the Eating Disorders Inventory-I (EDI-I; Garner et al., 1983). The EDI-I is a broadly utilized instrument with well-established reliability and validity (Garner et al., 1983).

#### Alexithymia

The Toronto Alexithymia Scale (TAS) was used to assess alexithymia, described as the inability to identify and describe emotions and tendency to minimize emotional experience by focusing attention externally to oneself (Bagby et al., 1994). We used the 20-item version of the TAS in this study, which includes three subscales: “Identifying,” or difficulty identifying feelings and distinguishing them from bodily sensations; “Describing,” or difficulty describing feelings to others; and “Externalizing,” or externally oriented thinking. The reliability and factorial validity for the TAS have been established previously (Bagby et al., 1994). Troop et al. (1995) examined the factor structure of the TAS with ED and HC groups, finding that ED groups were less able to identify their feelings compared to HCs. The TAS has demonstrated excellent internal consistency, good test–retest reliability, and good convergent and discriminant validity (Graeme et al., 1997).

#### Depression and Anxiety Symptoms

The Hospital Anxiety and Depression Rating Scale (HADS; Zigmond and Snaith, 1983) was used to assess the General psychiatric symptoms of anxiety and depression. This scale is a brief (14-item), self-report measure of anxiety and depression (Zigmond and Snaith, 1983). The HADS is a widely used measure in clinical practice and research (Herrmann, 1997) and has been used in non-clinical populations (Crawford et al., 2001).

### fMRI Stimuli

Three continuous blocks with 96 images per block of low energy (e.g., a bunch of English spinach) and high energy (e.g., a block of Cadbury’s chocolate) sweet and savory food, and neutral non-food objects (e.g., a brick office block and a door), were presented at 1-s intervals *via* dual video screen goggles equipped with eye tracking capability. A Rapid Serial Visual Presentation (RSVP) task was used to control for any potential attentional-related confounds. Participants were given a buzzer to hold in each hand during the scan and were asked to tap their index finger on the buzzers if they saw the same image twice in a row. The food images were further categorized within the low energy and high energy food images, in terms of evoking emotional responses of disgust, happiness, or fear. The images were created by author PH and in a previous study (McNamara et al., 2008), have been shown to evoke emotions in these categories in a sample of individuals with diagnosed eating disorders. Examples of food images in the different low energy emotive categories include: disgust—raw baby octopus; happy—a bowl of fresh cherries; and fear—fresh Birdseye chillies. Examples of food images in the high energy emotive categories include: disgust—a variety of raw sausages; happy—colorful iced cupcakes; and fear—whole BBQ ducks hanging by the neck.

A six-block design was used for presenting the images during the scan (three high/low energy food images × three emotions evoked). Each block took 16 s to complete in which 16 images of the same category of food and neutral, non-food images were presented. Images were presented in three runs of random order. Each run comprised six different categories including high and low energy food images in happiness, disgust, and fear categories. Imaging was conducted following a regular meal. Each fMRI scan took approximately 50 min to complete.

### fMRI Image Acquisition

Functional MRI data were captured on a GE GoldSeal 3.0T Signa HDxT (Software version 15M4, 8 channel array head coil) located at the Westmead Hospital’s Department of Radiology and Imaging. Images were all completed in sagittal orientation. fMRI runs were acquired using a gradient echo planar sequence (TR = 2 s, TE = 40 ms, 128 × 128 matrix, 240 mm FOV, 30 slices of 2 mm thick + 20% spacing). Prior to the first run, a T1-weighted sequence (spin-echo, 256 × 192 matrix, 21 slices of 4 mm thick + 1.5 mm spacing) was performed to obtain anatomical detail in the same slice planes used for fMRI. A high-resolution T1-weighted sequence was also acquired at the end of each participant’s scan [Inversion Recovery-prepared, Spoiled Gradient Refocused gradient echo (IRSPGR) 1 mm × 1 mm in-plane, 1 mm thick slices] followed by a B0 image.

### Data Analysis

Pre-processing and data analysis were performed *via* Statistical Parametric Mapping (SPM12) software (Wellcome Trust Centre for Neuroimaging, 2013). The SPM software package was designed for the analysis of functional brain imaging data and is a well-accepted software choice to test hypotheses about such data (Ashburner et al., 2013).

The first four volumes of each run were removed prior to analysis. Functional data were realigned within scanning runs to correct for head motion using a set of six rigid body transformations determined for each image. Using the echo planar imaging (EPI) template supplied with SPM12, each functional run was spatially normalized beginning with a local optimization of the 12 parameters of an affine transformation. These images were then smoothed with a 6 mm Gaussian filter. The blood-oxygen-level-dependent (BOLD) signal was analyzed within each run using a high-pass frequency filter, with a cutoff of 144 s. Correlations between scans and epochs were modeled by a standard hemodynamic response function at each voxel. Parameter estimates were obtained for each condition and participant to allow second-level random effects analysis of between-participant variability.

MATLAB R2017b (MathsWorks, 1998) was used in conjunction with SPM12 (Wellcome Trust Centre for Neuroimaging, 2013) to perform statistical analyses on fMRI data. First-level, or individual participant analysis, was completed by generating brain contrast images in each condition of the image stimuli (disgust low energy—neutral; fear low energy—neutral; and happy low energy—neutral) to determine the specific effects of the food image emotive categories. First-level contrasts were then used in second-level analyses.

Second-level, or between-group data analysis, was completed using a full factorial design with two groups (BEG and HC) and three values per group (low energy happy/fear/disgust) or (high energy happy/fear/disgust). Using the well-accepted and widely used fMRI thresholding guidelines published by Lieberman and Cunningham (2009), second-level analyses were completed using a combined statistical threshold of *p* < 0.005 and a voxel cluster size threshold of 10 contiguous voxels, which produces the ideal balance between Type I and Type II error rates. WFU_Pickatlas version 2.5 (Maldjian et al., 2003) was used to complete objective anatomical labeling for any clusters that reached significance. Exploratory whole-brain analyses were completed using the same thresholding guidelines described above (Lieberman and Cunningham, 2009).

Following exploratory analyses, six ROI’s were identified to investigate potential clinical correlates of aberrant activity. Beta values for individual subjects were extracted from each of the six ROIs using the SPM12 MarsBar toolbox (release 0.44; Brett et al., 2002), contrasting (BEG-HC) and (HC-BEG). Beta values were then correlated with clinical measures to establish any links. We have previously found that illness severity, measured by the frequency of objective and subjective binge eating episodes in the past month, was related to aberrant neural activity (Donnelly et al., 2018). Other clinical variables alongside the frequency of binge episodes, included for the purpose of beta value correlations were: frequency of purging; the EDE global score; HADS depression and anxiety subscales; EDI body image subscale; and TAS global score. Multiple linear regressions were conducted to assess whether either of the aforementioned clinical variables explained for significant variance in the ROI activity. The neural activity in the respective ROI was entered in each model as the dependent variable, while the clinical data were the independent variables. Unfortunately medications were not consistently recorded for each participant in this study so could not be included in the analysis.

## Results

### Sample Characteristics

Data from 19 females in the BEG and 19 HC were analyzed. In the BEG, 14 participants had a diagnosis of BN and five had a diagnosis of BED. The mean BMI of the BEG (25.04) and HC (22.98) did not differ significantly between the groups. The mean age was 26.63 years in the BEG and 21.74 years in the HC group, no significant difference between the mean age was found. These and other features are reported in Table 1.

To confirm the decision of grouping BN and BED groups together for the purpose of investigating neural activity in patients who binge eat, non-parametric independent samples Kruskal–Wallis tests were completed on weight and BMI. No statistically significant differences were found between individuals with a diagnosis of BN or BED for weight (*p* = 0.325) or BMI (*p* = 0.160).

As expected, the clinical group scored significantly higher on all subscales plus the global scale of the EDE-Q, and the HADS depression and anxiety scales. The BEG scored significantly higher on the TAS subscales of identifying and describing, while the externalizing subscale did not yield a significant difference. Levels of satiety pre- and post-fMRI did not differ between the groups.

### fMRI Data

Neural activation results contrasted between groups; BEG contrasted with HC (BEG-HC) and HC contrasted BEG (HC-BEG) are described below in order of low energy food images compared to neutral images for each of the different low energy food image conditions, followed by results of the high energy food images compared to neutral images for each of the high energy food image conditions. See Table 2 for results pertaining to neural activation in response to low energy food images across disgust, fear, and happy food image categories compared to neutral images, across groups. See Table 3 for results pertaining to high energy and neutral food images.

**Table 2:** Neural activation while viewing low energy disgust, fear, and happy food images compared to neutral images, across groups.

		MNI coordinates		
Group contrast^*^	Region (TD label, TD lobe)	k_E_	x	y	z	*p*
Low energy disgust > neutral BEG > HC	-	-	-	-	-	-
Low energy disgust > neutral HC > BEG
	**Culmen, CAL**	5,210	20	−36	−22	<0.001
	Sub-gyral, occipital lobe	2,148	−34	−68	−8	<0.001
	**Extra-nuclear, sub-lobar**	963	8	−65	−10	<0.001
	Precentral gyrus, frontal lobe	299	38	−2	30	<0.001
	Cuneus, occipital lobe	277	10	−88	22	<0.001
	MFG, frontal lobe	193	−44	40	−6	<0.001
	Cingulate gyrus, limbic system	149	−16	−6	48	<0.001
	Postcentral gyrus, parietal lobe	118	−10	−42	68	<0.001
	Sub-Gyral, Temporal Lobe	117	38	−12	−22	<0.001
Low energy fear > neutral BEG > HC						
	MOG, occipital lobe	296	−30	−70	6	<0.001
	**Culmen, CAL**	201	14	−46	−8	<0.001
	Postcentral gyrus, parietal lobe	113	46	−22	24	<0.001
	Sub-gyral, temporal lobe	96	38	−70	10	<0.001
	Parahippocampal gyrus, limbic system	39	−34	−32	−26	0.002
Low energy fear > neutral HC > BEG						
	SFG, frontal lobe	181	4	4	64	<0.001
	MFG, frontal lobe	137	32	50	26	<0.001
	**Tuber, cerebellum posterior lobe**	66	−22	−82	−38	<0.001
	IFG, frontal lobe	45	50	22	−2	<0.001
Low energy happy > neutral BEG > HC						
	Sub-gyral, temporal lobe	34	−28	−68	28	0.003
Low energy happy > neutral HC > BEG	-	-	-	-	-	-

TD, Talairach Daemon neurological database; MNI, montreal neurological Institute coordinates; k_E_, voxel size; CAL, cerebellum anterior lobe; MFG, middle frontal gyrus; MOG, middle occipital gyrus; SFG, superior frontal gyrus; and IFG = inferior frontal gyrus. Bold text indicates exploratory analyses.

* Group contrasts are abbreviated, for example, “Low energy disgust > neutral BEG > HC” which means low energy disgust food images compared to neutral images, with the BEG compared to HC in this condition. BEG, binge eating group comprising bulimia and binge eating disorder; and HC, healthy controls.

**Table 3:** Neural activation while viewing high energy disgust, fear, and happy food images compared to neutral images, across groups.

		MNI coordinates		
Group contrast^*^	Region (TD label, TD lobe)	k_E_	x	y	z	*p*
High energy disgust > neutral BEG > HC						
	-	-	-	-	-	-
High energy disgust > neutral HC > BEG						
	Posterior cingulate, limbic system	247	10	−58	4	<0.001
	**Third ventricle, sub-lobar**	121	0	−4	−12	<0.001
	Lingual gyrus, occipital lobe	100	−12	−60	−2	<0.001
	Sub-gyral, temporal lobe	96	−40	−74	10	<0.001
	MTG, temporal lobe	82	42	4	−34	<0.001
High energy fear > neutral BEG > HC						
	-	-	-	-	-	-
High energy fear > neutral HC > BEG						
	MFG, frontal lobe	96	10	2	66	<0.001
	IFG, frontal lobe	39	48	36	−8	0.002
High energy happy > neutral BEG > HC						
	-	-	-	-	-	-
High energy happy > neutral HC > BEG						
	Anterior cingulate, limbic system	56	−2	2	−6	<0.001

TD, Talairach Daemon neurological database; MNI, montreal neurological institute coordinates; k_E_, voxel size; MTG, middle temporal gyrus; MFG, middle frontal gyrus; and IFG, inferior frontal gyrus. Bold text indicates exploratory analyses.

* Group contrasts are abbreviated, for example, “High energy disgust > neutral BEG > HC” which means high energy disgust food images compared to neutral images, with the BEG compared to HC in this condition. BEG, binge eating group comprising bulimia and binge eating disorder; and HC, healthy controls.

### Low Energy Food Images

#### Low Energy Disgust Food Images Compared to Neutral Condition Images

##### BEG Compared to HC

Between-group analyses found no significant differences in neural activity in whole-brain or ROI analyses in the clinical group compared to HCs, while viewing images of low energy disgust foods in the exploratory or ROI tests.

##### HC Compared to BEG

In the HC-BEG group contrast, low energy disgust food images elicited significantly greater neural activity in the HCs in a large number of regions in exploratory analyses. For the ROI analyses, significantly greater neural activity was found in the HC’s in the sub-gyral (occipital lobe), middle temporal gyrus (temporal lobe), cingulate gyrus (limbic system), and postcentral gyrus (parietal lobe). See Table 2 for results.

#### Low Energy Fear Food Images Compared to Neutral Condition Images

##### BEG Compared to HC

In whole-brain analyses, the BEG demonstrated greater neural activity in a range of ROI’s in response to viewing low energy fear food images compared to HCs. The BEG had greater neural activity in the middle occipital gyrus (MOG; occipital lobe), sub-gyral (temporal lobe), parahippocampal gyrus (limbic system), and postcentral gyrus (parietal lobe; Table 2).

##### HC Compared to BEG

When viewing images of low energy fear foods, relative to the clinical group, the HC group demonstrated greater neural activity almost exclusively in the frontal lobe in exploratory analyses, with the addition of the tuber (cerebellum posterior lobe). In the ROI analyses, low energy fear food images provoked greater activity in the middle frontal gyrus (MFG), inferior frontal gyrus (IFG), and superior frontal gyrus (SFG; all frontal lobe; Table 2).

#### Low Energy Happy Food Images Compared to Neutral Condition Images

##### BEG Compared to HC

In whole-brain and ROI analyses, the low energy happy food images provoked significant neural activation only in the sub-gyral (temporal lobe) in the BEG, when compared to the HC group (Table 2).

##### HC Compared to BEG

Between-group analyses found no significant differences in neural activity in the HCs when compared to the BEG, while viewing images of low energy happy foods (Table 2).

#### Low Energy Food Images Condition Beta Value Correlation Analyses With Clinical Data

As seen in Table 2, a range of ROI’s was associated with both increased and decreased neural activity in binge eaters across the different low energy food image conditions. On examination of these results, a subset of ROI’s was identified with greater aberrant responding than other regions in binge eaters. Specifically, the occipital lobe, frontal lobe, and temporal lobe were *hypo*active in binge eaters, while looking at low energy food images in the disgust category; in the fear category, the occipital lobe and temporal lobe were *hyper*active while the frontal lobe was *hypo*active. Finally, the temporal lobe was again *hypo*active in response to food images in the happy category. Therefore, we calculated correlations between the clinical variables outlined above (frequency of objective or subjective binge episodes in the past month; frequency of purging episodes in the past month; EDE global score; HADS depression and anxiety subscales; EDI body image subscale; and TAS global score) and neural activity in all three ROI’s (occipital lobe, frontal lobe, and temporal lobe) in the disgust condition; in the occipital and temporal lobes in the fear condition; and, in the temporal lobe in the happy condition. To increase accuracy and robustness of the beta value correlation results with multiple tests being conducted, results were deemed significant after a Bonferroni correction of *p* = 0.0166 (0.05/3).

#### Beta Value Correlations: Low Energy Disgust Food Images Compared With Neutral Images

There were no statistically significant correlations with clinical variables (frequency of objective or subjective binge episodes in the past month; frequency of purging episodes per month; EDE global score; HADS depression and anxiety subscales; EDI body image subscale; and TAS global score) for the sub-gyral/occipital lobe cluster (k_E_ = 2,148). We note that one clinical variable approached significance, being frequency of OBEs over the past month (illness severity) in BEG participants (*r* = 0.558, *p* = 0.007). That is, there is a possible negative correlation between an increase of objective binge episodes and decreased activity in the sub-gyral/occipital lobe in response to low energy disgust food images. There were no statistically significant correlations between the decreased activity seen in the precentral gyrus/frontal lobe (k_E_ = 299) and any clinical variable. There was a statistically significant correlation in the sub-gyral/temporal lobe (k_E_ = 117) in relation to illness severity (*r* = −0.625, *p* = 0.002) at the Bonferroni-corrected level. That is, we found a significant negative correlation between frequency of objective binge episodes and hypoactivity in this ROI.

#### Beta Value Correlations: Low Energy Fear Food Images Compared to Neutral Images

The increased activity seen in the MOG/occipital lobe cluster (k_E_ = 299) was positively correlated with the frequency of both objective binge episodes (*r* = 0.565, *p* = 0.006) and subjective binge episodes (*r* = 0.553, *p* = 0.007) over the past month in BEG participants. For the sub-gyral/temporal lobe cluster (k_E_ = 96), there were no significant correlations with any clinical variable.

Lastly, there was decreased activity observed in the frontal lobe region in binge eaters relative to HCs (SFG, MFG, and IFG). For the present beta value correlational analyses, we extracted beta values from the SFG/frontal lobe as it had the greatest aberrant activity between the BEG and HCs (k_E_ = 181). There were no statistically significant correlations in this region.

#### Beta Value Correlations: Low Energy Happy Images Compared to Neutral Images

As with the low energy fear food images, increased activity was observed in the BEG in the sub-gyral/temporal lobe. No statistically significant correlations were found in this region.

### High Energy Food Images

#### High Energy Disgust Food Images Compared to Neutral Images

##### BEG Compared to HC

There were no whole-brain exploratory or ROI differences evoked in BEG participants compared to HC’s in this condition. See Table 3 for results.

##### HC Compared to BEG

Whole-brain exploratory analysis found the HCs had greater neural response to high energy disgust food images in the third ventricle (sub-lobar) compared to the BEG. In ROI analyses, high energy disgust food images led to significantly greater neural activation in the HC group, within the posterior cingulate (limbic system); lingual gyrus (occipital lobe); sub-gyral (temporal lobe); and middle temporal gyrus (temporal lobe).

#### High Energy Fear Food Images Compared With Neutral Images

##### BEG Compared to HC

The high energy fear food images evoked no significant differences in either whole-brain exploratory analyses or ROI analyses, in the BEG participants compared to HC’s.

##### HC Compared to BEG

In the HC group, there were no differences in whole-brain exploratory analyses. Neural activation in response to high energy fear food images was significantly greater in two areas of the frontal lobe in ROI analyses, the medial frontal gyrus, and the IFG.

#### High Energy Happy Food Images Compared to Neutral Condition Images

##### BEG Compared to HC

As with the previous categories, the high energy happy food images evoked no significantly different neural activation in the BEG compared to HC’s in either whole-brain exploratory or ROI analyses.

##### HC Compared to BEG

The high energy happy food images evoked no differences in the whole-brain exploratory analyses and greater activation in the anterior cingulate (limbic system) in the ROI analyses.

#### Beta Value Correlation Analyses With Clinical Data

As seen in Table 3, high energy food images evoked increased neural activity only in HC participants and not in the BE group, in a range of ROI’s. In the BEG, results demonstrate a profile of overall hyporesponsivity to high energy food images in ROI and whole-brain exploratory analyses. Three ROI’s were identified with higher neural response in the HC’s and decreased response in the BEG across all high energy food image categories, specifically the limbic system (disgust and happy high energy), temporal lobe (disgust high energy), and frontal lobe (fear high energy).

In line with the same analyses completed for the low energy foods data, to increase accuracy and robustness of the beta value correlation results with multiple tests being conducted, results were considered significant after a Bonferroni correction of *p* = 0.0166 (0.05/3). No significant correlations were found between frequency of binge episode in the past month (OBE or SBE); frequency of purging in the past month; EDE-Q global score; HADS depression and anxiety subscales; EDI body image subscale; or the TAS global score.

## Discussion

The aim of this study was to examine neural response during fMRI to low energy and high energy food images in individuals with BN or BED, compared to a HC group. Particular attention was given to whether the respective low/high energy food images stimuli evoked increased or decreased neural activity in the ROI’s we investigated. Consistent with our hypothesis, we found significantly different neural activity between the two groups, across low and high energy food conditions.

This study is the first to our knowledge, to complete separate analyses of the neural responses to low energy and high energy food images during fMRI, in individuals with recurrent binge eating symptoms in the context of BN or BED. This is an important contribution, as how the brain responds to high energy foods in individuals currently experiencing frequent and distressing binge episodes, had not been studied previously. Furthermore, the food images were additionally categorized into three emotive categories (disgust, fear, and happy) which added to the analyses completed. We also examined the role of clinical variables, including illness severity defined as the frequency of binge eating episodes and purging in the month preceding the fMRI scan, to investigate any association with neural activity. These data provide clarifying evidence regarding which different neural regions are implicated in disorder-relevant stimuli processing in individuals who binge eat. Furthermore, these data provide the first evidence that illness severity, defined by the frequency of binge episodes, is associated with how the brain responds to food stimuli in women who binge eat. The results of this paper are the first to report a pattern of neural deactivation in BN and BED in response to high energy food images when compared to HC’s.

### Low Energy Food Image Stimuli

In relation to examining differential activity between the BEG and HCs in neural regions reported in previous studies using food image stimuli, our results have identified a range of regions in exploratory and ROI analyses, a few that support previous research and others that are novel. Our fMRI data confirmed that, of the six ROI’s identified *via* exploratory analyses, five regions demonstrated significantly different responses between groups; being the frontal, occipital, parietal, and temporal lobes, and the cingulate gyrus.

We observed three ROI’s with differential neural activity in BEG participants during the low energy food image conditions: the occipital lobe, frontal lobe, and temporal lobe. In terms of the occipital lobe, which is involved with processing visual stimuli, we found that the BEG demonstrated significantly diminished activation of this region compared to HCs when looking at low energy disgust food images. This is consistent with the findings of Brooks et al. (2011), who found people with BN had deactivation in the occipital lobe when compared with HCs while viewing food images. A similar pattern of results has been previously reported (Mohr et al., 2011), wherein a BN group showed diminished occipital lobe activity when looking at own-body image distortions, compared to HCs. One possible explanation is that binge eaters disengage visually with this type of stimuli to avoid emotional discomfort.

An opposite pattern occurred in the occipital lobe when the BEG viewed low energy fear food images, with significantly greater activity in this region compared to HCs. This is a novel finding and as a way to understand this, in relation to the correlations between neural activity and clinical variables, our analyses found hyperactivity in the occipital lobe when viewing fear foods was related to increased illness severity or frequency of binge episodes. That is, as illness severity increased, activity in the occipital lobe did too. It remains unclear why the BEG had significantly greater activity in the occipital lobe while viewing fear foods, and diminished activity while viewing disgust foods. Uher et al. (2004) reported increased occipital lobe activation in response to food stimuli in their BN group compared to HC’s, observed in our BEG participants in response to low energy fear stimuli.

In terms of the frontal lobe, associated with planning, decision-making, behavioral inhibition, and cognition, we observed on overall pattern of diminished activity in the BEG, while viewing low energy food stimuli, both in the disgust and the fear food image categories. These results depart from previous studies that have reported greater activity in the frontal lobe region in response to food image stimuli in BN or BED participants; however, these studies used food images that did not separate high/low energy foods (Brooks et al., 2011) or, used only high-calorie and highly palatable foods (Schienle et al., 2009).

In the temporal lobe, broadly linked to cognition, emotion, learning, and memory, the BEG had greater activity compared to HCs in response to fear and happy low energy food images, while the HCs had greater activity in the disgust condition compared to the BEG. In relation to the latter results, we went on to find a significant negative correlation between the hypoactivity in the temporal lobe of the BEG in the disgust condition and illness severity. This specific finding is in line with that of Brooks et al. (2011), who reported deactivation of the temporal gyrus in their BN sample in response to food images during fMRI.

### High Energy Food Image Stimuli

The novel findings of these data were that in response to high energy food images, the BEG demonstrated a consistently diminished neural response to high energy food stimuli. Specifically, results demonstrated significantly decreased activity in four of the six ROI’s (frontal, temporal, occipital lobes, and limbic system), no significantly increased activity in any ROI, and no regions of increased activity in exploratory whole-brain analyses. Furthermore, the overall profile of diminished neural activity seen in the BEG, compared to the HC’s, occurred in each of the three emotive categories of high energy food images—disgust, fear, and happy. Unlike the low energy food images, correlational analyses with the high energy food image fMRI data found no relationship between the decreased neural activity in the BEG and clinical variables of illness severity, measured by the frequency of OBE’s and SBE’s in the past month; purging frequency in the past month; EDE global score; HADS depression and anxiety subscales; EDI body image subscale; and TAS global score.

One possible explanation for the findings in the high energy food image condition is that the diminished response to these types of foods seen in the BEG relative to the HC’s may be due to hypofunctioning of neural regions associated with reward and gustatory circuitry. In one fMRI study examining neural activation in response to real and anticipated food intake in females with BN and HC’s, Bohon and Stice (2011) found that women with BN demonstrated decreased activation in several regions of the brain associated with reward (insula, MFG, and precentral gyrus). The results of their study highlight that the diminished activity of these regions in BN may be linked to hypofunctioning of the neural reward system, which in turn may explain why this group binge eat to compensate for the deficit in reward (Bohon and Stice, 2011). In the present study, we observed significantly decreased activation in the MFG in response to high energy fear food images in the BEG relative to the HC’s. The MFG is known to be activated in response to taste in previous studies (Kringelbach et al., 2004).

Related to the findings of decreased neural activity in reward regions, a previous fMRI study found a glucose solution consumed during imaging evoked significantly less activation in regions of the brain associated with reward in individuals who had recovered from BN compared to HC’s (Frank et al., 2006). The prefrontal cortex is one region implicated in reward processing and BN patients have previously demonstrated weaker involvement of this area, which may reflect the deficits in control associated with compulsive binge eating (Giel et al., 2011). However, not all previous food stimuli studies are in line with our results in terms of the specific regions of decreased activity. In the first ED study of neural response to food image stimuli to include individuals with BN, Uher et al. (2004) found the BN group differed to AN and HC groups by having decreased activation in the anterior and lateral prefrontal cortex in response to food.

The finding of decreased activity in the ACC in the BEG relative to the HC’s in response to high energy happy food images is of note. The ACC forms part of the broader cortico-striato-thalamo-cortical (CSTC) loops that relay information from cortical, to subcortical and then back to cortical regions. Corticostriatal circuits play a regulatory role in motivation and impulse control, and changes in inhibitory control have been thought to be implicated in increased binge eating behavior (Voon, 2015). The frontostriatal circuit, believed to be involved in self-regulation and reward-based learning, lies within the broader CSTC (Berner and Marsh, 2014). That the BEG had significantly diminished activity in the ACC when viewing foods that are most consistent with the highly palatable, sweet foods that are consumed during a BE, lends support to the theory that functional deficits in BN and BED in the frontostriatal circuits may behaviorally translate into urges being unchecked, for example, to continue eating or engage in compensatory behaviors (Berner and Marsh, 2014).

Disgust sensitivity has been investigated in the field of EDs due to the features of this emotion (e.g., shame) that are explicitly linked to the emotions associated with eating for those with an ED. In one emotion study of individuals with AN and BN, drive for thinness and bulimic symptoms were related to higher disgust sensitivity to food (Troop et al., 2000). In a further study, current and recovered ED patients reported higher disgust toward food and the body (Harvey et al., 2002). One explanation may be that because of elevated disgust sensitivity, individuals with ED’s visually disengage during neuroimaging to avoid potentially uncomfortable emotional states. With the high energy disgust food images evoking significantly decreased activation in the BEG (in both low energy and high energy papers) compared to the HC’s, we can postulate the neural response to disgust in a non-ED person triggers a complex interplay of regions associated with visual processing (occipital lobe), emotion, memory and motivation (limbic system), integration of sensory information (parietal lobe), integration of sensory information, memory and language (temporal lobe), and executive functions (frontal lobe). Hypoactivity in the third ventricle in the BEG compared to HCs while viewing the high energy disgust images is also of note. Uher and Treasure (2005) examined the relationship between brain lesions and eating disordered behavior in 54 papers. Twenty-three of these reported lesions in the third ventricle and hypothalamus prompted a range of eating disorder behaviors including binge eating and purging. A cautious explanation for the finding of reduced activity in this region in the BEG is that the third ventricle may be implicated in the perception of, and decision-making regarding food, which could explain why this group are more indiscriminate toward food consumption, leading to a sense of feeling out of control.

### Summarizing Our Results: A Neural Model of Binge Eating

The findings in the current study underscore the observation that there is a lack of neural response in the BEG to both high/low energy and fear, disgust, happy foods. In relation to clinically meaningful results, our data has highlighted a pattern of diminished neural responding to most food stimuli, irrespective of high or low energy foods, in individuals who binge eat. One possibility is that there is a mechanism of reduced satiation demonstrated by the diminished neural responding to food stimuli in fMRI, together with an imbalance of the fronto-striatal reward system and control-related circuit abnormalities underpinning the intensity and frequency of binge eating. We have found that the frequency of binge episodes was associated with how the brain responds to food stimuli in women who binge eat, which presents a clinically important maintaining factor of this illness, which can be difficult to treat.

## Limitations and Conclusion

Although this study provides novel findings regarding diminished neural activity in a sample of BN and BED participants in response to low and high energy food images, limitations must be noted. The sample size in each group (*n* = 19) exceeded power requirements; however, increasing the sample may have improved our ability to detect wider results in relation to beta value correlations with other clinical variables beyond illness severity. In order to increase homogeneity in the sample, we did not recruit males for this study despite BN and BED occurring in women and men, although at lower rates in men (Hay et al., 2017). Medications were not consistently recorded; therefore, we could not include this variable in the correlational analyses. A further limitation is we relied on the self-report of participants to advise researchers that they had consumed a normal meal prior to attending their fMRI appointment. Previous neuroimaging studies of ED participants have highlighted the tendency of this clinical group to fast for longer than requested prior to neuroimaging (Uher et al., 2004; Schienle et al., 2009). Neural response is effected by hunger and satiety, so we recommend future studies improve this specific method. Further to this point, making a broader assessment of the “fed state” of the participants in the preceding 24 h would be ideal, due to blood sugar levels and hunger being dependent on this state and the possible impact on neural response. We encourage future studies to follow the methodology of Bohon and Stice (2011), who asked participants to consume a standardized snack 1 h prior to fMRI. Additionally, the design of the study is cross-sectional; hence to determine whether any of the neural differences between groups predicts BN or BED, a longitudinal design must be implemented.

To build on the results of this study, we recommend researchers carefully consider the design and stimuli used during future fMRI studies. Considering the impact differing study designs and methodologies are likely to have on our knowledge of the neural response to food in BN and BED, it would be recommended for future researchers to replicate an improved version of the current design, firstly by separating high and low energy food stimuli during fMRI and secondly, by using food images that have been validated in this and the preceding study (Donnelly et al., 2019). Secondly, and as mentioned previously, although correlational analyses in this paper did not find a relationship between aberrant neural activity in the BEG and severity of illness, this relationship has been reported in a systematic review (Donnelly et al., 2018), and the neural response to low energy food images study preceding this investigation (Donnelly et al., 2019). Lastly, ongoing neuroimaging research is warranted to continue to contribute to the development of neuroscience-based models of binge eating (Frank, 2013), BN and BED, which will in turn contribute to improvements in effective treatments for individuals seeking treatment for these disorders. Pairing pharmacological interventions or other neuromodulator interventions that are known to enhance cognitive control and patient outcomes in this clinical group, with cognitive behavioral therapy or other evidence-based psychological therapies (Hilbert et al., 2019) could offer a promising treatment plan for individuals who continue to compulsively binge eat (Berner et al., 2011; Berner and Marsh, 2014; Bello and Yeomans, 2017). Longitudinal research would also inform the question of how much the brain responses’ may be due to state (illness) vs. trait (inherent vulnerability) states of people with binge eating.

While our findings are mixed, the present study contributes to increased understanding of the neuroimaging profile of BN and BED. Our finding of the relationship between illness severity and differential neural activity in response to low energy food images warrants further exploration; also, it is reasonable to state that this finding is of clinical relevance. Providing a clearer picture of the neurobiological differences seen in people with BN and BED compared to HCs will lead to increased understanding of the cognitive processes that characterize the responses to disorder-relevant stimuli. Importantly, by assisting individuals to develop an understanding of the nuanced ways in which their brain responds to food in the ill-state of an eating disorder characterized by frequent binge episodes, we believe it will reduce the stigma for individuals and increase engagement. Additionally, using this information in relation to developing cognitive strategies that will assist people with BN or BED to engage differently with food stimuli and in turn, gradually change their emotive and attentional response, we believe this would be beneficial in terms of improving treatment outcomes. Because normalizing eating is a core component of psychological treatment for any individual with an ED, it is essential to improve understanding of how patient groups differentially respond to food on a neural basis, as the foundation of treatment.

## Data Availability Statement

The raw data supporting the conclusions of this article will be made available by the authors, without undue reservation, and in compliance with appropriate ethical clearance.

## Ethics Statement

The studies involving human participants were reviewed and approved by Sydney Children’s Hospital human research ethics committee (HREC) number HREC/13/SCHN/385. Written informed consent to participate in this study was provided by the participant and where applicable by the participants’ legal guardian/next of kin.

## Author Contributions

BD conducted the literature review, data curation and data analysis, compiled the first draft, and undertook subsequent edits. MW conducted higher level design, data curation and data analysis, contributed to the first draft, and assisted with subsequent edits. NF conducted data curation and data analysis, contributed to the first draft, and assisted with subsequent edits. AP oversighted the imaging and reporting, contributed to the first draft, and assisted with subsequent edits. ST, JR, and IC undertook a supervisory role, contributed to the first draft, and assisted with subsequent edits. SM, MK, and SC advised on design, facilitated recruitment, contributed to the first draft, and assisted with subsequent edits. PH undertook a supervisory role, contributed to design, data curation and data analysis, contributed to the first draft, and assisted with subsequent edits. All authors contributed to the article and approved the submitted version.

## Funding

Funding was provided to PH from WSU for scanning costs.

## Conflict of Interest

The authors declare that the research was conducted in the absence of any commercial or financial relationships that could be construed as a potential conflict of interest.

## Publisher’s Note

All claims expressed in this article are solely those of the authors and do not necessarily represent those of their affiliated organizations, or those of the publisher, the editors and the reviewers. Any product that may be evaluated in this article, or claim that may be made by its manufacturer, is not guaranteed or endorsed by the publisher.

## References

[ref1] Amianto F. D'Agata F. Lavagnino L. Caroppo P. Abbate-Daga G. Righi D.. (2013). Intrinsic connectivity networks within cerebellum and beyond in eating disorders. Cerebellum 12, 623–631. doi: 10.1007/s12311-013-0471-1, PMID: 23553468

[ref2] APA (2013). Diagnostic and Statistical Manual of Mental Disorders. 5th *Edn*. Arlington, VA: APA.

[ref3] Appelhans B. (2009). Neurobiobehavioral inhibition of reward-driven feeding: implications for dieting and obesity. Obesity 17, 640–647. doi: 10.3389/fnbeh.2014.00395, PMID: 19165160

[ref4] Ashburner J. Barnes G. Chen C. Daunizeau J. Flandin G. Friston K.. (2013). SPM8 Manual. UCL, 12 Queen Square, London WC1N 3BG, UK: Functional Imaging Laboratory, Wellcome Trust Centre for Neuroimaging, Institute of Neurology, University College London.

[ref5] Bagby R. M. Parker J. D. A. Taylor G. J. (1994). The twenty-item Toronto alexithymia scale. Item selection and cross-validation of the factor structure. J. Psychosom. Res. 38, 23–32. doi: 10.1016/0022-3999(94)90005-1, PMID: 8126686

[ref6] Beglin S. Fairburn C. (1992). Evaluation of a new instrument for the detection of eating disorders in community samples. Psychiatry Res. 44, 191–201. doi: 10.1016/0165-1781(92)90023-V, PMID: 1289917

[ref7] Bello N. T. Yeomans B. L. (2017). Safety of pharmacotherapy options for bulimia nervosa and binge eating disorder. Expert Opin. Drug Saf. 17, 17–23. doi: 10.1080/14740338.2018.1395854, PMID: 29053927 PMC6095708

[ref8] Berner L. A. Bocarsly M. E. Hoebel B. G. Avena N. M. (2011). Pharmacological interventions for binge eating: lessons from animal models, current treatments, and future directions. Curr. Pharm. Des. 17, 1180–1187. doi: 10.2174/138161211795656774, PMID: 21492094

[ref9] Berner L. Marsh R. (2014). Frontostriatal circuits and the development of bulimia nervosa. Front. Behav. Neurosci. 8:395. doi: 10.3389/fnbeh.2014.00395, PMID: 25452718 PMC4233924

[ref10] Bohon C. Stice E. (2011). Reward abnormalities among women with full and subthreshold bulimia nervosa: a functional magnetic resonance imaging study. Int. J. Eat. Disord. 44, 585–595. doi: 10.1002/eat.20869, PMID: 21997421 PMC3111910

[ref11] Brett M. Anton J.-L. Valabregue R. Poline J.-B. (2002). Region of interest analysis using an SPM toolbox (abstract). *Paper Presented at the 8th International Conference on Functional Mapping of the Human Brain;* June 2-6. Sendai, Japan.

[ref12] Brooks S. J. O’Daly O. G. Uher R. Friederich H. C. Giampietro V. Brammer M.. (2011). Differential neural responses to food images in women with bulimia versus anorexia nervosa. PLoS One 6:e22259. doi: 10.1371/journal.pone.0022259, PMID: 21799807 PMC3140495

[ref13] Crawford J. R. Henry J. D. Crombie C. Taylor E. P. (2001). Brief report normative data for the HADS from a large non-clinical sample. Br. J. Clin. Psychol. 40, 429–434. doi: 10.1348/014466501163904, PMID: 11760618

[ref14] Desmond J. E. Glover G. H. (2002). Estimating sample size in functional MRI (fMRI) neuroimaging studies: statistical power analyses. J. Neurosci. Methods 118, 115–128. doi: 10.1016/S0165-0270(02)00121-8, PMID: 12204303

[ref01] Donnelly B. A. (2019). A Neuroimaging Examination of Binge Eating in Bulimia Nervosa and Binge Eating Disorder (Doctoral dissertation). University of Sydney. Available at: https://ses.library.usyd.edu.au/handle/2123/21357 (Accessed March 5, 2022).

[ref15] Donnelly B. Touyz S. Hay P. Burton A. Russell J. Caterson I. (2018). Neuroimaging in bulimia nervosa and binge eating disorder: a systematic review. J. Eat. Disord. 6:3. doi: 10.1186/s40337-018-0187-1, PMID: 29468065 PMC5819247

[ref16] Fairburn C. G. (2008). Cognitive Behavior Therapy and Eating Disorders. New York: Guildford Press.

[ref17] Foroughi N. Madden S. Clarke S. Kohn M. Donnelly B. Touyz S.. (2018). Do emotional responses to food images differ within different types of eating disorders? Australas. Psychiatry 25, 128–133. doi: 10.1177/1039856218789790, PMID: 30113878

[ref18] Frank G. K. W. (2013). Altered brain rewards circuits in eating disorders: chicken or egg? Curr. Psychiatry Rep. 15:396. doi: 10.1007/s11920-013-0396-x, PMID: 23963630 PMC3888645

[ref19] Frank G. K. Wagner A. Achenbach S. McConaha C. Skovira K. Aizenstein H.. (2006). Altered brain activity in women recovered from bulimic-type eating disorders after a glucose challenge: a pilot study. Int. J. Eat. Disord. 39, 76–79. doi: 10.1002/eat.20210, PMID: 16254868

[ref20] Garcia-Garcia I. Narberhaus A. Marques-Iturria I. Garolera M. Radoi A. Segura B.. (2013). Neural responses to visual food cues: insights from functional magnetic resonance imaging. Eur. Eat. Disord. Rev. 21, 89–98. doi: 10.1002/erv.2216, PMID: 23348964

[ref21] Garner D. M. Olmstead M. P. Polivy J. (1983). Development and validation of a multidimensional eating disorder inventory for anorexia nervosa and bulimia. Int. J. Eat. Disord. 2, 15–34. doi: 10.1002/1098-108X(198321)2:2<15::AID-EAT2260020203>3.0.CO;2-6

[ref22] Giel K. E. Teufel M. Friedrich H. Hautzinger M. Enck P. Zipfel S. (2011). Processing of pictorial food stimuli in patients with eating disorders—a systematic review. Int. J. Eat. Disord. 44, 105–117. doi: 10.1002/eat.20785, PMID: 20127931

[ref23] Graeme J. Taylor R. Bagby M. Parker J. D. A. (1997). Disorders of Affect Regulation: Alexithymia in Medical and Psychiatric Illness. Cambridge UK: Cambridge University Press, 359.

[ref24] Harvey T. Troop N. A. Treasure J. L. Murphy T. (2002). Fear, disgust and abnormal eating attitudes: a preliminary study. Int. J. Eat. Disord. 32, 213–218. doi: 10.1002/eat.10069, PMID: 12210664

[ref25] Hay P. Katsikitis M. (2014). Emotional responses to images of food in adults with an eating disorder: a comparative study with healthy and clinical controls. Eat. Behav. 15, 371–374. doi: 10.1016/j.eatbeh.2014.04.016, PMID: 25064283

[ref26] Hay P. Mitchison D. Collado A. E. L. Gonzales-Chica D. A. Stocks N. Touyz S. (2017). Burden and health-related quality of life of eating disorders, including avoidant/restrictive food intake disorder (ARFID), in the Australian population. J. Eat. Disord. 5:21. doi: 10.1186/s40337-017-0149, PMID: 28680630 PMC5494787

[ref27] Herrmann C. (1997). International experiences with the hospital anxiety and depression scale—a review of validation data and clinical results. J. Psychosom. Res. 42, 17–41. doi: 10.1016/S0022-3999(96)00216-4, PMID: 9055211

[ref28] Hilbert A. Petroff D. Herpertz S. Pietrowsky R. Tuschen-Caffier B. Vocks S.. (2019). Meta-analysis of the efficacy of psychological and medical treatments for binge-eating disorder. J. Consult. Clin. Psychol. 87, 91–105. doi: 10.1037/ccp0000358, PMID: 30570304

[ref29] Kessler R. Hutson P. H. Herman B. K. Potenza M. N. (2016). The neurobiological basis of binge-eating disorder. Neurosci. Biobehav. Rev. 63, 223–238. doi: 10.1016/j.neubiorev.2016.01.013, PMID: 26850211

[ref30] Kringelbach M. L. de Araujo I. T. B. Rolls E. T. (2004). Taste-related activity in the human dorsolateral prefrontal cortex. NeuroImage 21, 781–788. doi: 10.1016/j.neuroimage.2003.09.063, PMID: 14980581

[ref31] Lavagnino L. Arnone D. Cao B. Soares J. C. Selvaraj S. (2016). Inhibitory control in obesity and binge eating disorder: a systematic review and meta-analysis of neurocognitive and neuroimaging. Neurosci. Biobehav. Rev. 68, 714–726. doi: 10.1016/j.neubiorev.2016.06.041, PMID: 27381956

[ref32] Lieberman M. D. Cunningham W. A. (2009). Type I and type II error concerns in fMRI research: re-balancing the scales. Soc. Cogn. Affect. Neurosci. 4, 423–428. doi: 10.1093/scan/nsp052, PMID: 20035017 PMC2799956

[ref33] Mahler P. Guastavino J. M. Jacquart G. Strazielle C. (1993). An unexpected role of the cerebellum: involvement in nutritional organization. Physiol. Behav. 54, 1063–1067. doi: 10.1016/0031-9384(93)90325-A, PMID: 8295941

[ref34] Maldjian J. A. Laurienti P. J. Kraft R. A. Burdette J. H. (2003). An automated method for neuroanatomic and cytoarchitectonic atlas-based interrogation of fMRI data sets. NeuroImage 19, 1233–1239. doi: 10.1016/S1053-8119(03)00169-1, PMID: 12880848

[ref35] MathsWorks (1998). MatlabR2017b. Menlo Park, CA: Addison-Wesley.

[ref36] McClelland J. Dalton B. Kekic M. Bartholdy S. Campbell I. C. Schmidt U. (2016). A systematic review of temporal discounting in eating disorders and obesity: behavioural and neuroimaging findings. Neurosci. Biobehav. Rev. 71, 506–528. doi: 10.1016/j.neubiorev.2016.09.024, PMID: 27693228

[ref37] McNamara C. Chur-Hansen A. Hay P. (2008). Emotional responses to food in adults with an eating disorder: a qualitative exploration. Eur. Eat. Disord. Rev. 16, 115–123. doi: 10.1002/erv.810, PMID: 17680589

[ref38] Mohr H. M. Roder C. Zimmermann J. Hummel D. Negele A. Grabhorn R. (2011). Body image distortions in bulimia nervosa: investigating body size overestimation and body size satisfaction by fMRI. NeuroImage 56, 1822–1831. doi: 10.1016/j.neuroimage.2011.02.069, PMID: 21362488

[ref39] Mond J. M. Hay P. J. Rodgers B. Owen C. (2006). Eating disorder examination questionnaire (EDE-Q): norms for young adult women. Behav. Res. Ther. 44, 53–62. doi: 10.1016/j.brat.2004.12.003, PMID: 16301014

[ref40] Phillipou A. Rossell S. L. Castle D. J. (2014). The neurobiology of anorexia nervosa: a systematic review. Aust. N. Z. J. Psychiatry 48, 128–152. doi: 10.1177/0004867413509693, PMID: 24194589

[ref41] Rodríguez S. M. J. Lameiras M. Fernandez M. C. Vila J. (2007). Dyscontrol evoked by erotic and food images in women with bulimia nervosa. Eur. Eat. Disord. Rev. 15, 231–239. doi: 10.1002/erv.724, PMID: 17676693

[ref42] Santel S. Baving L. Krauel K. Munte F. Rotte M. (2006). Hunger and satiety in anorexia nervosa: fMRI during cognitive processing of food pictures. Brain Res. 1114, 138–148. doi: 10.1016/j.brainres.2006.07.045, PMID: 16919246

[ref43] Schienle A. Schafer A. Hermann A. Vaitl D. (2009). Binge-eating disorder: reward sensitivity and brain activation to images of food. Biol. Psychiatry 65, 654–661. doi: 10.1016/j.biopsych.2008.09.028, PMID: 18996508

[ref44] Sheehan D. Janavs J. Baker R. Harnett-Sheehan K. Knapp E. Sheehan M. (2005). Mini International Neuropsychiatric Interview, English Version 5.0.0 USA.

[ref45] Skunde M. Walther S. Simon J. J. Wu M. Bendszus M. Herzog W.. (2016). Neural signature of behavioural inhibition in women with bulimia nervosa. J. Psychiatry Neurosci. 41, E69–E78. doi: 10.1503/jpn.150335, PMID: 27575858 PMC5008924

[ref46] Troop N. A. Murphy F. Bramon E. Treasure J. (2000). Disgust sensitivity in eating disorders: a preliminary investigation. Int. J. Eat. Disord. 27, 446–451. doi: 10.1002/(SICI)1098-108X(200005)27:4<446::AID-EAT9>3.0.CO;2-W, PMID: 10744851

[ref47] Troop N. A. Schmidt U. Treasure J. (1995). Feelings and fantasy in eating disorders: a factor analysis of the Toronto alexithymia scale. Int. J. Eat. Disord. 18, 151–157. doi: 10.1002/1098-108X(199509)18:2<151::AID-EAT2260180207>3.0.CO;2-E, PMID: 7581417

[ref48] Uher R. Brammer M. J. Murphy T. Campbell I. C. Ng V. W. Williams S. C. R.. (2003). Recovery and chronicity in anorexia nervosa: brain activity associated with differential outcomes. Biol. Psychiatry 54, 934–942. doi: 10.1016/S0006-3223(03)00172-0, PMID: 14573322

[ref49] Uher R. Murphy T. Brammer M. J. Dalgleish T. Phillips M. L. Ng V. W.. (2004). Medial pre-frontal cortex activity associated with symptom provocation in eating disorders. Am. J. Psychiatr. 161, 1238–1246. doi: 10.1176/appi.ajp.161.7.1238, PMID: 15229057

[ref50] Uher R. Treasure J. (2005). Brain lesions and eating disorders. J. Neurol. Neurosurg. Psychiatry 76, 852–857. doi: 10.1136/jnnp.2004.048819, PMID: 15897510 PMC1739667

[ref51] Voon V. (2015). Cognitive biases in binge eating disorder: the hijacking of decision making. CNS Spectr. 20, 566–573. doi: 10.1017/S1092852915000681, PMID: 26594850 PMC4697296

[ref52] Wellcome Trust Centre for Neuroimaging (2013). Statistical Parametric Mapping (SPM12): Institute of Neurology, University College London.

[ref53] Wierenga C. Ely A. Bischoff-Grethe A. Bailer U. Simmons A. Kaye W. (2014). Are extremes of consumption in eating disorders in eating disorders related to an altered balance between reward and inhibition? Front. Behav. Neurosci. 8:410. doi: 10.3389/fnbeh.2014.00410, PMID: 25538579 PMC4260511

[ref54] Zhu J. N. Wang J. J. (2008). The cerebellum in feeding control: possible function and mechanism. Cell. Mol. Neurobiol. 28, 469–478. doi: 10.1007/s10571-007-9236-z, PMID: 18027085 PMC11515829

[ref55] Zigmond A. S. Snaith R. P. (1983). The hospital and anxiety and depression scale. Acta Psychiatr. Scand. 67, 361–370. doi: 10.1111/j.1600-0447.1983.tb09716.x 6880820

[ref56] Zunker C. Peterson C. B. Crosby R. D. Cao L. Engel S. G. Mitchell J. E.. (2011). Ecological momentary assessment of bulimia nervosa: does dietary restriction predict binge eating? Behav. Res. Ther. 49, 714–717. doi: 10.1016/j.brat.2011.06.006, PMID: 21764036 PMC3168587

